# Multivariate genome wide association and network analysis of subcortical imaging phenotypes in Alzheimer’s disease

**DOI:** 10.1186/s12864-020-07282-7

**Published:** 2020-12-29

**Authors:** Xianglian Meng, Jin Li, Qiushi Zhang, Feng Chen, Chenyuan Bian, Xiaohui Yao, Jingwen Yan, Zhe Xu, Shannon L. Risacher, Andrew J. Saykin, Hong Liang, Li Shen, for the Alzheimer’s Disease Neuroimaging Initiative

**Affiliations:** 1 School of Computer Information & Engineering, Changzhou Institute of Technology, Changzhou, 213032 China; 2 College of Automation, Harbin Engineering University, Harbin, 150001 China; 3 School of Computer Science, Northeast Electric Power University, Jilin, 132012 China; 4 Department of Biostatistics, Epidemiology and Informatics, University of Pennsylvania Perelman School of Medicine, Philadelphia, PA 19104 USA; 5 Department of Radiology and Imaging Sciences, Indiana University School of Medicine, Indianapolis, IN 46202 USA; 6 Department of BioHealth Informatics, Indiana University School of Informatics and Computing, Indianapolis, IN 46202 USA

**Keywords:** Brain imaging, Multivariate gene-based genome-wide analysis, iPINBPA network analysis, Consensus modules

## Abstract

**Background:**

Genome-wide association studies (GWAS) have identified many individual genes associated with brain imaging quantitative traits (QTs) in Alzheimer’s disease (AD). However single marker level association discovery may not be able to address the underlying biological interactions with disease mechanism.

**Results:**

In this paper, we used the MGAS (Multivariate Gene-based Association test by extended Simes procedure) tool to perform multivariate GWAS on eight AD-relevant subcortical imaging measures. We conducted multiple iPINBPA (integrative Protein-Interaction-Network-Based Pathway Analysis) network analyses on MGAS findings using protein-protein interaction (PPI) data, and identified five Consensus Modules (CMs) from the PPI network. Functional annotation and network analysis were performed on the identified CMs. The MGAS yielded significant hits within *APOE, TOMM40* and *APOC1* genes, which were known AD risk factors, as well as a few new genes such as *LAMA1*, *XYLB*, *HSD17B7P2*, and *NPEPL1*. The identified five CMs were enriched by biological processes related to disorders such as Alzheimer’s disease, Legionellosis, Pertussis, and Serotonergic synapse.

**Conclusions:**

The statistical power of coupling MGAS with iPINBPA was higher than traditional GWAS method, and yielded new findings that were missed by GWAS. This study provides novel insights into the molecular mechanism of Alzheimer’s Disease and will be of value to novel gene discovery and functional genomic studies.

## Background

Alzheimer’s disease (AD) is a debilitating and highly heritable disease with great complexity in its genetic contributors [1]. Genome-wide association studies (GWAS) of AD or AD biomarkers have been performed at the single-nucleotide polymorphism (SNP) level [2–4] as well as at the higher level (e.g., gene, pathway and/or network) [5–8]. It is widely recognized that AD has a complicated genetic mechanism involving multiple genes. Different combinations of functionally related variants in genes and pathways may interact to produce the phenotypic outcomes in AD, single SNP-level and gene-level GWAS results are unlikely to completely reveal the underlying genetic mechanism in AD. GWAS have greatly facilitated the identification of genetic markers (e.g., single nucleotide polymorphisms or SNPs) associated with brain imaging quantitative traits (QTs) in AD [9, 10]. As a complex disease, it is highly likely that AD is influenced by multiple genetic variants [11, 12]. The identified single-SNP-single-QT associations typically have small effect sizes. To bridge this gap, exploring single-SNP-multi-QT associations may have the potential to increase statistical power and identify meaningful imaging genetic associations. With this observation, we employ the MGAS (Multivariate Gene-based Association test by extended Simes procedure) tool [13] to perform multivariate GWAS on eight AD-relevant subcortical imaging measures.

In addition, biological interactions may be important in contributing to intermediate imaging QTs and overall disease outcomes [14]. Network-based analysis guided by biologically relevant connections from public databases provides a powerful tool for improved mechanistic understanding of complex disorders [15–18]. Considering that the etiology of AD might depend on functional protein-protein interaction (PPI) network, we conduct multiple iPINBPA (integrative protein-interaction-network-based pathway analysis) [19] network analyses on MGAS findings using the PPI data, and identify Consensus Modules (CMs) based on the iPINBPA discoveries. Functional annotation and network analysis are subsequently performed on the identified CMs.

**Fig. 1:**
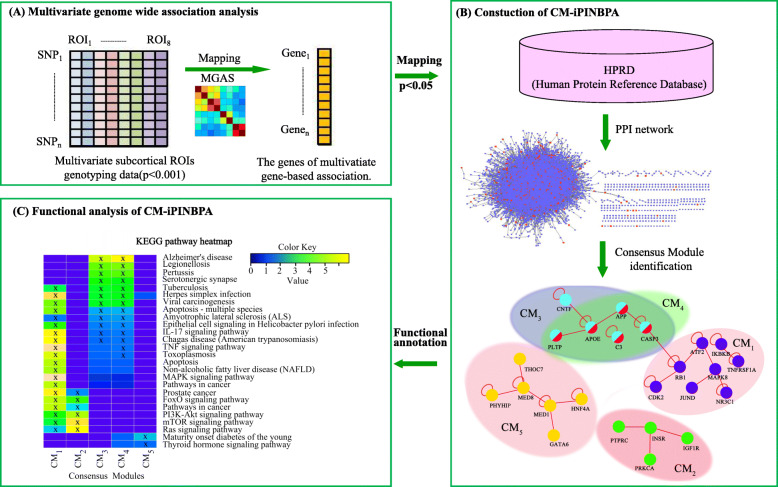
An overview of the proposed analysis framework. **a** Multivariate genome wide association analysis of eight subcortical imaging measures. **b** Network-based analysis of MGAS findings using the CM-based network strategy. **c** Functional enrichment analysis of the identified consensus modules (CMs)

In order to enhance the ability to recognize the aggregation effect of multiple SNPs, it may be desirable to perform association analysis at the SNP set (or gene) level rather than at a single SNP level. This paper aims to reveal the relationship between genetic markers and multiple phenotypes, improve statistical power, and find GWAS missing results by MGAS. Network analysis could provide meaningful biological relationships to help interpret GWAS data to further study the genetic mechanism of AD. A schematic framework of our analysis is shown in Fig. 1.

## Results

### Participant characteristics

**Fig. 2:**
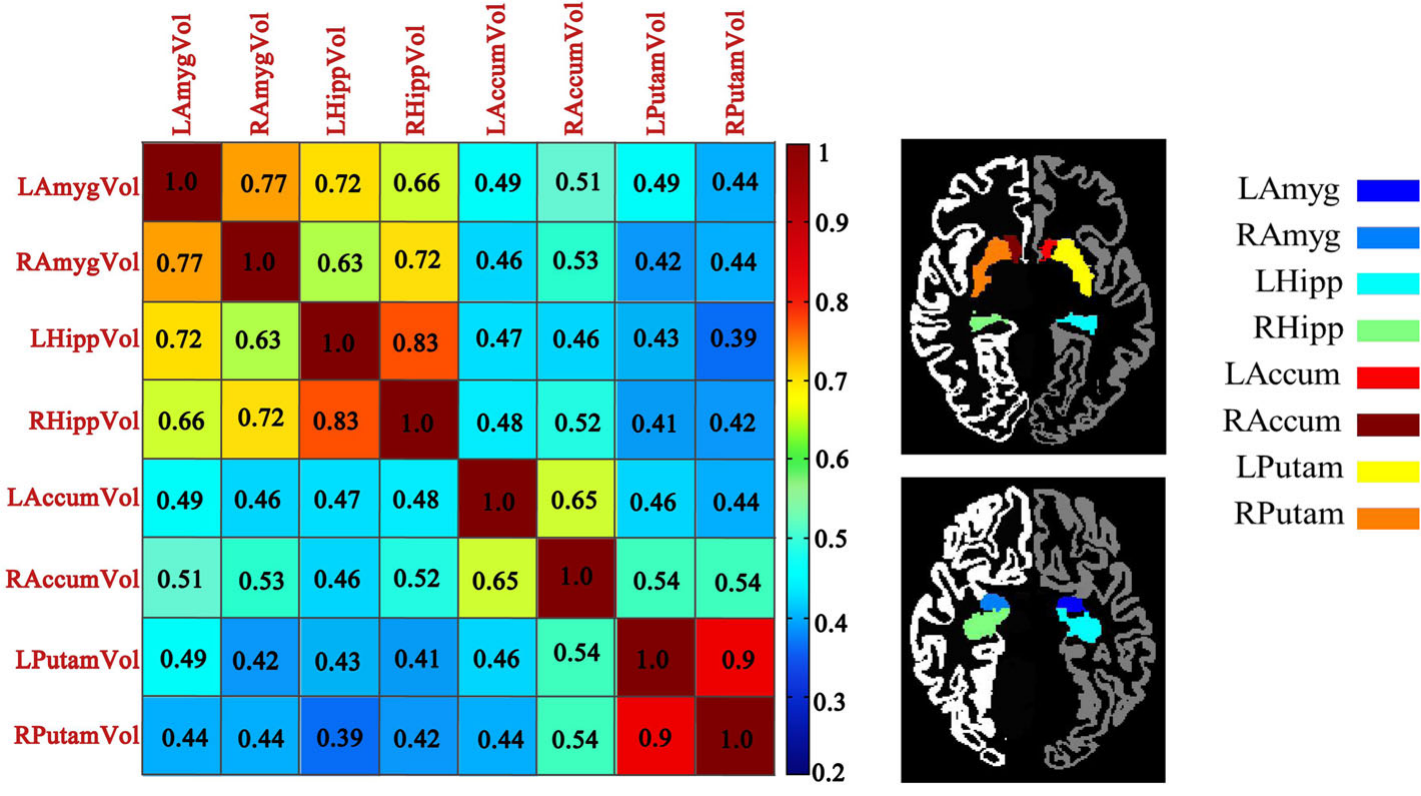
Phenotypic correlations between 8 subcortical volumes traits, volumes

The subjects (*N* = 866) consisted of 467 males (53.9%) and 399 females (46.1%) aged 48–91 years. Shown in Table 1 are the demographic and clinical characteristics of these subjects stratified by five diagnostic groups. There is no significant difference on the *APOE* e4 status in the five diagnostic groups. Significant differences are observed in gender (*p* = 0.035) and education (*p* = 0.037). Age is significantly different across the five groups (*p* < 0.001). Furthermore, eight neuroimaging phenotypes (LAmygVol,RAmygVol,LHippVol,RHippVol, LAccumVol,RAccumVol,LPutamVol,RPutamVol; see Table 2) show the significant difference across the five diagnostic groups (*p <* 0.001). Shown in Fig. 2 is the correlation matrix of these eight phenotypes. The correlation between LHippVol and RHippVol (*r =* 0.83) and that between LPutamVol and RPutamVol (*r =* 0.90) are among the highest.

**Table 1:** Demographic information and total number of participants involved in each analysis

	CN (*N* = 183)	SMC (*N* = 95)	EMCI (*N* = 281)	LMCI (*N* = 177)	AD (*N* = 130)	*p-*values
Age (years)	74.29(6.01)	72.20(5.67)	71.35(7.30)	71.87(7.98)	74.56(8.07)	*p <* 0.001
Gender(M/F)	92/91	39/56	159/122	99/78	78/52	0.035
Education (years)	15.78(2.69)	16.81(2.55)	16.07(2.66)	16.31(2.89)	15.78(2.69)	0.037
APOE e4 allele prensent	62(34%)	37(39%)	108 (38%)	64(36%)	45 (35%)	0.161
**LAmygVol** **(i.e., QT for GWAS)**	**1377.2 (230.40)**	**1434.48(202.03)**	**1387.06(257.16)**	**1258.07(286.96)**	**1081.93 (229.42)**	***p <*** **0.001**
**RAmygVol** **(i.e., QT for GWAS)**	**1425.05 (221.11)**	**1494.02 (210.39)**	**1450.03 (255.77)**	**1327.93 (282.28)**	**1178.03 (240.54)**	***p <*** **0.001**
**LHippVol** **(i.e., QT for GWAS)**	**3626.23 (497.63)**	**3730.98 (529.42)**	**3573.44 (558.02)**	**3243.94 (635.34)**	**2912.15 (518.08)**	***p <*** **0.001**
**RHippVol** **(i.e., QT for GWAS)**	**3679.88 (500.54)**	**3824.23 (487.64)**	**3663.93 (535.43)**	**3319.32 (641.19)**	**2985.63 (540.11)**	***p <*** **0.001**
**LAccumVol** **(i.e., QT for GWAS)**	**463.51 (100.88)**	**481.37 (92.49)**	**474.07 (93.96)**	**447.91 (101.37)**	**417.88 (96.74)**	***p <*** **0.001**
**RAccumVol** **(i.e., QT for GWAS)**	**490.44 (95.69)**	**506.76 (93.38)**	**508.65 (107.70)**	**474.41 (105.41)**	**429.57 (96.56)**	***p <*** **0.001**
LCaudVol	3442.57 (505.06)	3411.83 (518.23)	3477.88 (572.20)	3460.31 (518.58)	3429.95 (698.19)	0.0851
RCaudVol	3583.43 (528.08)	3539.56 (545.67)	3640.80 (623.54)	3588.41 (540.35)	3577.51 (697.23)	0.608
LPallVol	1602.48 (204.00)	1592.57 (196.97)	1633.63 (211.56)	1587.56 (220.94)	1584.27 (231.88)	0.101
RPallVol	1413.36 (189.35)	1434.79 (177.66)	1444.59 (192.61)	1433.18 (202.08)	1414.99 (217.48)	0.444
**LPutamVol** **(i.e., QT for GWAS)**	**4788.04 (636.56)**	**4788.21 (687.42)**	**4939.46 (750.31)**	**4733.41 (671.80)**	**4479.92 (688.84)**	***p <*** **0.001**
**RPutamVol** **(i.e., QT for GWAS)**	**4586.97 (599.25)**	**4584.27 (604.70)**	**4708.87 (752.76)**	**4522.97 (669.07)**	**4327.07 (687.91)**	***p <*** **0.001**
LThalVol	6060.0 (714.50)	6064.96 (868.69)	6072.55 (604.95)	6054.47 (745.24)	5953.05 (718.64)	0.592
RThalVol	6081.58 (684.84)	6056.69 (722.49)	6176.78 (663.50)	6128.47 (732.28)	5980.53 (750.51)	0.098

*AD* Alzheimer’s disease, *ADNI* Alzheimer’s Disease Neuroimaging Initiative, *CDR–SOB* clinical dementia rating–sum of boxes, *CN* cognitively normal, *SMC* significant memory concern, *EMCI* early mild cognitive impairment, *LMCI* late mild cognitive impairment

Number (%) or mean (s.d.) is shown in each entry. *P-*values are computed using one-way ANOVA (*except for gender using chi-square test)

**Table 2:** 14 FreeSurfer subcortical ROIs

Phenotype ID	Description	Region
LAmygVol	the volume of the left amygdala	Subcortical (temporal)
RAmygVol	the volume of the right amygdala	
LHippVol	the volume of the left hippocampus	
RHippVol	the volume of the right hippocampus	
LAccumVol	the volume of the left accumbens	Subcortical (striatum/basal ganglia)
RAccumVol	the volume of the right accumbens	
LCaudVol	the volume of the left caudate	
RCaudVol	the volume of the right caudate	
LPallVol	the volume of the left pallidum	
RPallVol	the volume of the right pallidum	
LPutamVol	the volume of the left putamen	
RPutamVol	the volume of the right putamen	
LThalVol	the volume of the left thalamus	Subcortical (thalamus)
RThalVol	the volume of the right thalamus

### Multivariate genome wide association study

**Fig. 3:**
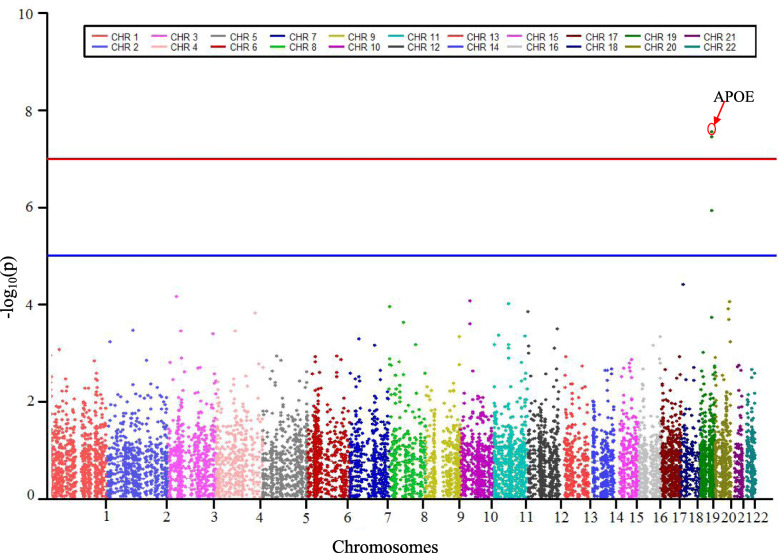
A Manhattan plot showing the gene-level *p* values in multivariate GWAS study of 8 subcortical volumes. The blue line corresponds to *p* = 10^− 5^; the red line corresponds to *p =* 10^− 7^

In multivariate genome wide association study (MGAS) [13, 20], the top SNP hit is rs769449 from the *APOE* and *TOMM40* region (*p* = 1.19E-09) (Table 3). According to the hypothesis that genes are the functional units in biology [15, 21], multivariate gene-level association *p*-values were also obtained by MGAS which combines *p-*value information in regressing univariate phenotypes on common SNPs. Figure 3 shows the Manhattan plot of the gene-based MGAS results. Using Bonferroni corrected *p-*value of 0.05 as the threshold, three genes (*APOE, TOMM40, APOC1*) were significantly associated with the studied eight subcortical measures. Table 4 shows that the top 10 gene-level findings identified by MGAS, where *APOE* (*p* = 2.77E-08), *TOMM40* (*p* = 3.49E-08), and *APOC1* (*p* = 2.09E-06) are the well-known AD risk regions. *LAMA1* (*p* = 3.79E-05) was reported to encode the laminin alpha subunit associated with late onset AD in the Amish [22]. *HSD17B7P2* (*p* = 8.40E-05) was reported to play an important role in brain development [23]. The other five gene-level findings in top 10 are *XYLB, NPEPL1, CYP24A1, OR5B2 and MIR7160.*

**Table 3:** The top 10 SNPs identified by MGAS

SNP	Position	P_**SNP**_	Chr	Gene	P_MGAS_
rs769449	45,410,002	1.19E-09	19	APOE	2.77E-08
rs405509	45,408,836	2.01E-04	19	APOE	2.77E-08
rs439401	45,414,451	1.99E-01	19	APOE	2.77E-08
rs584007	45,416,478	2.25E-01	19	APOE	2.77E-08
rs445925	45,415,640	2.62E-01	19	APOE	2.77E-08
rs769449	45,410,002	1.19E-09	19	TOMM40	3.49E-08
rs2075650	45,395,619	2.19E-06	19	TOMM40	3.49E-08
rs157582	45,396,219	8.54E-05	19	TOMM40	3.49E-08
rs1160985	45,403,412	6.88E-06	19	TOMM40	3.49E-08
rs405509	45,408,836	2.01E-04	19	TOMM40	3.49E-08

P_SNP_ is the *p-*value of top 10 snp identified by MGAS; Chr: Chromosome; P_MAGS_ is the *p-*value of top 10 gene identified by MGAS

**Table 4:** The top 10 FDR corrected genes identified by MGAS in 8 subcortical ROIs

NO.	Gene	P_MGAS_	Chr	SNP	P_LAmygVol_	P_RAmygVol_	P_LHippVol_	P_RHippVol_	P_LAccumVol_	P_RAccumVol_	P_LPutamVol_	P_RPutamVol_
1	APOE	2.77E-08	19	rs769449	***3.98E-06***	1.38E-04	**5.77E-07**	**9.52E-09**	1.58E+ 00	1.48E+ 00	2.11E+ 00	2.44E+ 00
2	TOMM40	3.49E-08	19	rs769449	***3.98E-06***	1.38E-04	**5.77E-07**	**9.52E-09**	1.58E+ 00	1.48E+ 00	2.11E+ 00	2.44E+ 00
3	APOC1	1.18E-06	19	rs4420638	3.09E-05	3.46E-05	***7.70E-06***	**5.23E-07**	6.99E-01	1.34E+ 00	3.06E+ 00	2.71E+ 00
4	LAMA1	3.79E-05	18	rs656734	2.62E-05	**8.64E-07**	3.11E-04	5.53E-04	5.64E+ 00	2.45E+ 00	4.27E+ 00	5.26E+ 00
5	XYLB	6.72E-05	3	rs196376	1.80E-02	6.61E-04	8.64E-05	***6.63E-06***	2.06E-02	5.68E-01	9.22E-01	7.13E-02
6	HSD17B7P2	8.40E-05	10	rs12221164	3.42E+ 00	4.05E+ 00	5.92E+ 00	5.09E+ 00	1.16E-04	1.02E-01	7.86E+ 00	7.93E+ 00
7	NPEPL1	8.63E-05	20	rs2426778	5.96E-01	3.89E+ 00	7.38E-01	2.17E+ 00	7.32E+ 00	2.09E-02	2.13E-05	3.39E-03
8	OR5B2	9.40E-05	11	rs11229440	1.62E+ 00	8.20E-01	3.90E-01	1.05E+ 00	1.67E+ 00	8.30E-03	2.22E-04	9.52E-05
9	MIR7160	1.10E-04	8	rs6558595	8.22E-01	7.69E+ 00	3.07E-01	1.13E+ 00	8.50E-02	1.68E+ 00	3.49E-05	3.67E-04
10	CYP24A1	1.20E-04	20	rs3787555	2.10E-01	4.75E+ 00	3.64E+ 00	2.19E+ 00	7.80E+ 00	1.75E+ 00	1.71E-01	9.55E-01

P_MAGS_ is the *p-*value of top 10 genes identified by MGAS; Chr: Chromosome; P_LAmygVol_ is the *p-*value of top 10 genes associated to LAmygVol; P_RAmygVol_ is the *p-*value of top 10 genes associated to RAmygVol; P_LHippVol_ is the *p-*value of top 10 genes associated to LHippVol; P_RHippVol_ is the *p-*value of top 10 genes associated to RHippVol; P_LAccumVol_ is the *p-*value of top 10 genes associated to LAccumVol; P_RAccumVol_ is the *p-*value of top 10 genes associated to RAccumVol; P_LPutamVol_ is the *p-*value of top 10 genes associated to LPutamVol; P_RPutamVol_ is the *p-*value of top 10 genes associated to RPutamVol, Bold font indicates *p-*value < 0.000001, Italic font indicates *p-*value< 0.00001

### Consensus modules

Consensus modules (CMs) were constructed based on our previous work [24]. To search for subnetworks in the multivariate GWAS finding, we ran iPINBPA ten times by varying the random seed value from 1 to 10. Table 5 shows the top 5 subnetworks identified in each run, including the Dice’s coefficient value with the most similar modules in other runs. Compared with the standard iPINBPA method, our CM-based network strategy was designed to identify more reliable modules across multiple runs.

**Table 5:** The characteristics of the identified consensus modules in 10 iPINBPA runs

CM	RunA	Ta: the top subnetwork in RunA. Sb: the most similar subnetwork to Ta in RunB										
		RunB	1	2	3	4	5	6	7	8	9	10
1	TN11	Rank of Sb in RunB	TN_11_	TN_12_	TN_13_	TN_14_	TN_15_	TN_16_	TN_17_	TN_18_	TN_19_	TN_1,10_
		DC(x, y)	**1**	**0.87**	**0.63**	**0.38**	**0.87**	**0.53**	**0.44**	**0.57**	**0.92**	**0.51**
2	TN21	Rank of Sb in RunB	TN_21_	TN_22_	TN_23_	TN_24_	TN_25_	TN_26_	TN_27_	TN_28_	TN_29_	TN_2,10_
		DC(x, y)	**1**	**0.77**	**0.58**	**0.71**	**0.95**	**0.59**	**0.47**	**0.7**	**0.77**	**0.38**
3	TN31	Rank of Sb in RunB	TN_31_	TN_32_	TN_33_	TN_34_	TN_45_	TN_36_	TN_37_	TN_38_	TN_49_	TN_4,10_
		DC(x,y)	**1**	**0.89**	**0.89**	**1**	**0.74**	**0.74**	**0.77**	**0.89**	**0.89**	**0.44**
4	TN41	Rank of Sb in RunB	TN_41_	TN_32_	TN_33_	TN_34_	TN_45_	TN_36_	TN_37_	TN_38_	TN_49_	TN_3,10_
		DC(x,y)	**1**	**0.61**	**0.61**	**0.7**	**0.96**	**0.67**	**0.67**	**0.7**	**0.7**	**0.36**
5	TN51	Rank of Sb in RunB	TN_51_	TN_62_	TN_53_	TN_74_	TN_75_	TN_86_	TN_67_	TN_88_	TN_89_	TN_4,10_
		DC(x, y)	**1**	**1**	**1**	**1**	**1**	**1**	**1**	**1**	**0.96**	**0.52**

**Fig. 4:**
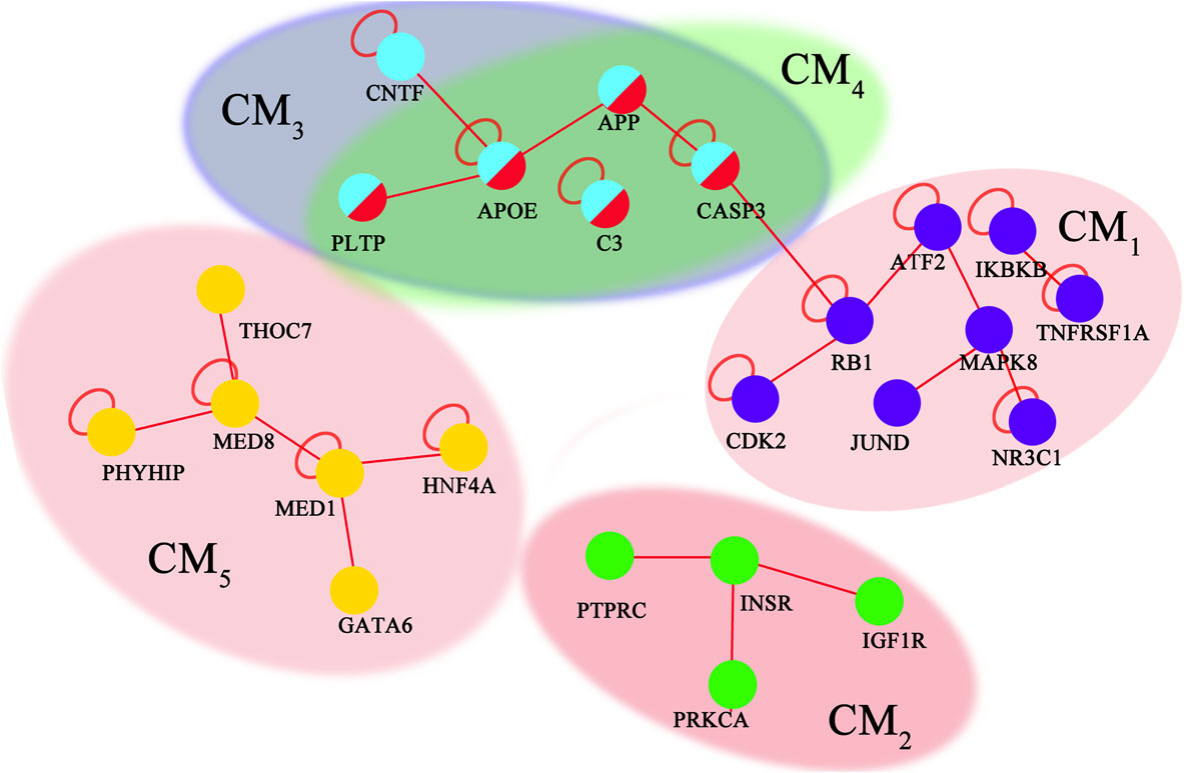
Consensus modules identified by CM-based network strategy. Different CMs are showed by different colors. The blue color indicates genes in CM_1_; green color indicates genes in CM_2_; cyan color indicates genes in CM_3_; red color indicates genes in CM_4_; and yellow color indicates genes in CM_5_.The genes appearing in multiple CMs have multiple colors. For example, the genes APOE, APP, CASP3, C3, PLTP are in both CM_3_ and CM_4_

For the overlapping subnetworks, five unique CMs were identified (Fig. 4). CM_1_ contains eight genes, including *MAPK8, ATF2, TNFRSF1A, JUND, NR3C1, RB1, IKBKB,* and *CDK2*. CM_2_ contains four genes, including *IGF1R, PRKCA, INSR,* and *PTPRC*. CM_3_ contains six genes, including *APP, APOE, CASP3, C3, PLTP,* and *CNTF*. CM_4_ contains five genes, including *APP, APOE, CASP3, C3,* and *PLTP.* CM_5_ contains six genes, including *MED8, GATA6, HNF4A, MED1, THOC7,* and *PHYHIP.* The individual genes in the CMs might not demonstrate a direct statistical significance. All the genes in an identified module have a collective effect on the studied QTs, and thus have the potential to provide valuable information about the underlying biology.

### Pathway analysis of consensus modules

**Fig. 5:**
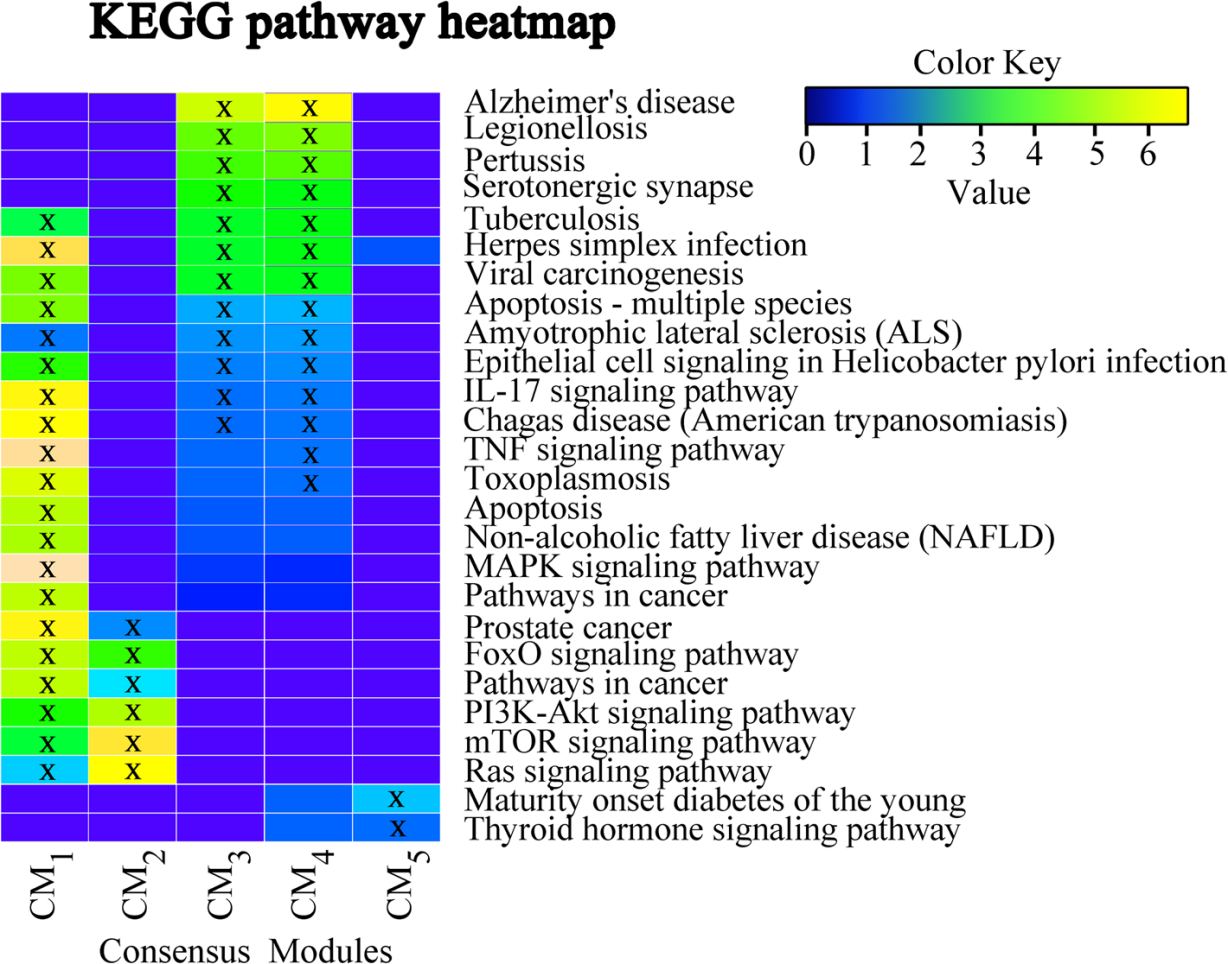
Functional annotation of the five identified consensus modules (CM_1_-CM_5_) using KEGG pathways. The five consensus modules were treated as five gene sets, and went through pathway enrichment analysis based on the KEGG pathway database. The enrichment results at a nominal statistical threshold of *p* < 0.05 are shown. -log10(p) values are color-mapped and displayed in the heat map. Heat map blocks labeled with “x” reach the nominal significance level of *p* < 0.05. Only top enrichment findings are included in the heat map, and so each row (pathway) has at least one “x” block

In our work, we hypothesize that the identified trait prioritized CMs with high replication might have strong functional associations with the studied subcortical volume phenotypes. We clustered the relevant pathways for five CMs and plotted a heat map to summarize the relationships between these pathways and CMs (Fig. 5). Figure 5 shows that Alzheimer’s disease, Apoptosis, TNF signaling pathway, Herpes simplex infection, MAPK signaling pathway are the pathways significantly enriched by one or more CMs [25]. We also observe that CM_1_, CM_3_, CM_4_ enriched many interesting pathways. In particular, CM_3_ demonstrates the strongest functional association with AD (*p* = 4.94E-05).

## Discussion

In this work, we performed multivariate genome wide association study (MGAS) of eight AD-relevant subcortical ROIs, using 866 samples in the ADNI database. To the best of our knowledge, this is the first MGAS on the quantitative traits of eight subcortical ROIs. In our MGAS, we confirmed associations at multiple genes previously associated with AD, such as *APOE* (*p* = 2.77E-08, rs769449), *TOMM40* (*p* = 3.49E-08, rs769449), *APOC1* (*p* = 1.18E-06, rs4420638), as well as identified a few novel associations shown in Table 4. Table 4 also shows that the associations to individual subcortical QTs (e.g. *APOE*, *TOMM40*, *APOC1*: associated to LAmygVol, RAmygVol, LHippVol and RHippVol) have a range of different significances.

*XYLB* (*p* = 6.72E-05, rs196376) had been reported to be associated with neurological diseases such as ischemic stroke [26]. We observed that this gene is associated to RAmygVol, LHippVol and RHippVol. *LAMA1* (*p* = 3.79E-05, rs656734) encodes one of the alpha 1 subunits of Laminin, which has been demonstrated to be expressed in the hippocampal neuronal cell layers [27]. *NPEPL1* was confirmed to be a potential direct target of miR-19a in a breast cancer study [28] and miR-19a was up-regulated in primary motor cortex and hippocampus in the brain of amyotrophic lateral sclerosis mice at late disease stage [29]. In our study, we found that *NPEPL1* was associated to LPutamVol (P_LPutamVol_ = 2.13E-05) and RPutamVol (P_RPutamVol_ = 3.39E-03). In Table 4, the hippocampus and amygdala volumes were associated with multiple genes. While *CYP24A1* was associated with none of eight studied QTs, it was identified in MGAS to have an overall association with all eight QTs. The statistical efficacy of MGAS of the detected gene associations appears to be more powerful than univariate phenotype models. Given that *OR5B2* and *MIR7160* have not been reported to be related to AD or AD related biomarkers, it warrants further investigation to examine their roles on AD in independent cohorts.

Because we found that different subnetworks could be identified by using different random seed values, we present the consensus modules discovered by an enhanced iPINBPA strategy. The genes for the CMs might not show a direct individual statistical significance but demonstrated a collected effect on the studied phenotypes. We assessed the significance of each identified consensus module (Table 6). CM_1_ (*Score* = 3.32, *p* = 9.00E-04) contains totally 8 genes, including KEGG AD genes *TNFRSF1A*. CM_2_ (*Score* = 1.41, *p* = 0.16) contains totally 4 genes without reaching the significance level. CM_3_ (*Score* = 4.37, *p* = 1.24E-05) contains 6 genes, including KEGG AD genes *APOE APP,* and *CASP3*. CM_4_ (*Score* = 4.32, *p* = 1.56E-05) contains 5 genes, including KEGG AD genes *APOE, APP*, and *CASP3*. CM_5_ (*Score* = 1.89, *p* = 5.88E-02) contains 6 genes with a marginal significance. The genes in the significant CMs warrant further investigation. The consensus module strategy applied to the iPINBPA framework yielded more stable results than the standard iPINBPA.

**Table 6:** The properties of Consensus Modules identified from the PPI network

Consensus Module	Nodes	Score	***P-***value
CM_1_	MAPK8, ATF2, TNFRSF1A, JUND, NR3C1, RB1, IKBKB, CDK2	3.32	9.00E-04
CM_2_	IGF1R, PRKCA, INSR, PTPRC	1.41	1.59E-01
CM_3_	APP, APOE, CASP3, C3, PLTP, CNTF	4.37	1.24E-05
CM_4_	C3, PLTP, APP, APOE, CASP3	4.32	1.56E-05
CM_5_	MED8, GATA6, HNF4A, MED1, THOC7, PHYHIP	1.89	5.88E-02

Scores were computed by using the adjusted network score

The intersection of CM_3_ and CM_4_ yielded five genes, including *APP, APOE, CASP3, C3,* and *PLTP*. The *C3* gene was shown to contribute to the pathogenesis of demyelinating disease by directly or indirectly chemoattracting encephalitogenic cells to the CNS [30]. The *PLTP* gene was reported to play an important role in Aβ metabolism and it is an interesting topic to further elucidate functions of *PLTP* in AD susceptibility. Table 7 shows the top ten pathways enriched by the intersection genes. Among these genes, the *APP, APOE,* and *CASP3* genes are known AD risk factors. Several significant pathways were observed, including Alzheimer’s disease (*p*-value = 2.50E-05, FDR = 1.54E-03), Legionellosis (*p-*value = 1.90E-04, FDR = 7.03E-03), Pertussis (*p-*value = 3.63E-04, FDR = 8.40E-03), Serotonergic synapse (*p-*value = 8.02E-04, FDR = 1.28E-02), Tuberculosis(*p-*value = 2.00E-03, FDR = 1.92E-02), Herpes simplex infection (*p-*value = 2.13E-03, FDR = 1.92E-02) and so on. It has been reported that Legionella pneumonphila, one species of Legionella, is an intracellular microorganism that causes Legionellosis. This type of pulmonary infection is usually associated with neurological dysfunction [31]. Serotonergic neurotransmission and synapse activity are highlighted as primary pathological factors in neuropsychiatric symptoms [32, 33]. Pertussis toxin inhibits the apoptosis and DNA synthesis caused by FAD APP mutants which precedes FAD APP-mediated apoptosis in neurons and inhibition of neuronal entry into the cell cycle inhibits the apoptosis [34]. Apoptotic pathways and DNA synthesis are activated in neurons in the brains of individuals with AD.

**Table 7:** The pathway of genes appearing in all five consensus modules

NO.	Pathway	*p-*value	FDR	Genes
1	Alzheimer’s disease	2.50E-05	1.54E-03	CASP3, APOE, APP
2	Legionellosis	1.90E-04	7.03E-03	CASP3, C3
3	Pertussis	3.63E-04	8.40E-03	CASP3, C3
4	Serotonergic synapse	8.02E-04	1.28E-02	CASP3, APP
5	Tuberculosis	2.00E-03	1.92E-02	CASP3, C3
6	Herpes simplex infection	2.13E-03	1.92E-02	CASP3, C3
7	Viral carcinogenesis	2.51E-03	1.92E-02	CASP3, C3
8	Apoptosis - multiple species	1.32E-02	2.94E-02	CASP3
9	Amyotrophic lateral sclerosis (ALS)	2.03E-02	3.92E-02	CASP3
10	*Staphylococcus aureus* infection	2.23E-02	4.12E-02	C3

Due to the limited number of samples available to us, in this work we were only able to perform a discovery study. In the future, when more data become available replication studies in independent cohorts warrant investigation to validate the identified CMs.

## Conclusion

In this study, we performed MGAS analysis to explore the multivariate imaging genetic association effects for a set of AD-related subcortical measures. In addition, we conducted the iPINBPA network analysis to discover consensus modules related to these imaging phenotypes from a protein-protein interaction network. The MGAS analysis identified several genes associated with the studied imaging phenotypes, including *APOE, TOMM40, APOC1, LAMA1, XYLB, HSD17B7P2* and others. The statistical power of coupling MGAS with iPINBPA was higher than traditional GWAS method, and yielded findings missed by GWAS. In this work, we reported top five consensus modules based on MGAS results. Network-based analysis can take into account information on biological relationships to interpret GWAS data. Our results suggested several susceptible genes and network modules for further investigation and replication to better understand the genetic mechanism of Alzheimer’s Disease.

## Methods

### Subjects and data

Data used in the preparation of this article were obtained from the Alzheimer’s Disease Neuroimaging Initiative (ADNI) database (adni.loni.usc.edu). The ADNI was launched in 2003 as a public-private partnership, led by Principal Investigator Michael W. Weiner, MD. The primary goal of ADNI has been to test whether serial magnetic resonance imaging (MRI), positron emission tomography (PET), other biological markers, and clinical and neuropsychological assessment can be combined to measure the progression of mild cognitive impairment (MCI) and early Alzheimer’s disease (AD). For up-to-date information, see www.adni-info.org.

Baseline 3 T MRI scans, demographic information, and diagnosis for the ADNI-1 and ADNI-GO/2 cohorts were downloaded [35]. MRI scans were analyzed using FreeSurfer version 5.1 for brain segmentation. We examined the volume measures of 14 subcortical ROIs; see Tables 1-2. We performed analysis of variance (ANOVA) to evaluate the diagnostic effect on 14 volume measures. Using the significance level of *p* < 0.05, we focused on the volume measures of eight subcortical ROIs (i.e., LAmygVol, RAmygVol, LHippVol, RHippVol, LAccumVol, LPutamVol, RPutamVol; see Table 2) in subsequent genetic association studies.

Genotyping data of both ADNI-1 and ADNI-GO/2 cohorts were downloaded, and then quality controlled and combined as described in [36]. A total of 866 non-Hispanic Caucasian participants with both complete subcortical imaging measurements and genotyping data were included in the study. The study sample (*N* = 866) included 183 cognitively normal (CN), 95 significant memory concern (SMC), 281 early MCI (EMCI), 177 late MCI (LMCI) and 130 AD subjects. The demographic and clinical characteristics of participants, stratified by the diagnosis, are shown in Table 1.

### Multivariate genome wide association study

GWAS was performed to examine the main effects of 563,980 SNPs on eight subcortical measures as quantitative traits (QTs). Linear regression model was performed using PLINK to examine the association between each SNP-QT pair (https://www.cog-genomics.org/plink2) [37]. An additive genetic model was tested with age, gender and brain volume as covariates. We computed the correlation matrix (8 × 8 matrix) for the QT data containing eight imaging phenotypes (Fig. 2). We applied the MGAS (Multivariate Gene-based Association test by extended Simes procedure) tool to all 563,980 SNPs and examined their multivariate gene-based associations with eight imaging QTs [13]. A Manhattan plot was generated using R (http://www.r-project.org) to visualize the gene-level MGAS results for our work (Fig. 3).We obtained one multivariate gene-based *p*-value P_MGAS_ as follows.

$$ {P}_{MGAS}=\min \left(\frac{q_e{p}_j}{q_{ej}}\right) $$

Here, *q*_*e*_ represents the effective number of *p*-values within a gene, *q*_*ej*_ represents the effective number of *p-*values among the top *j p-*values where *j* runs from 1 to 8 × 563,980, and *p*_*j*_ represents the *j*-th *p-*value in the list of ordered *p-*values. P_MGAS_ is the smallest weighted *p-*value within a gene associated with the null hypothesis that none of the eight phenotypes are related to the 563,980 SNPs within the gene, and the alternative hypothesis that at least one of the eight phenotypes is related to at least one of the 563,980 SNPs. We identified 1386 genes with *p-*value< 0.05 [13, 38].

### Identifying consensus models using iPINBPA

This study used the protein-protein interaction (PPI) data from the Human Protein Reference Database (HPRD, http://www.hprd.org) [39], containing 9617 proteins and 39,240 interactions. Gene-level *p-*values obtained from MGAS of subcortical imaging phenotypes were mapped to the PPI network. Then the network was followed by an iPINBPA (integrative protein-interaction-network-based pathway analysis) procedure [19] to identify enriched PPI network modules. Consensus modules (CMs) were identified using the following approach based on our prior study [24].

Briefly, building on our prior study, we focus on analyzing the top 5 subnetworks (TN_1_, TN_2_, TN_3_, TN_4_, TN_5_) in each iPINBPA run. Let *TN*_*ij*_ be the top *i*-th subnetwork identified in the *j*-th run, where *i* ∈ {1, 2, …, 5} and *j* ∈ {1, 2, ...10}. We first find *SN*_*n*_*(TN*_*ij*_*)*, which is the most similar subnetwork to *TN*_*ij*_ in the *n*-th run, where *n* ∈ {1, 2, …, 10}\{*j*}. Clearly, we have.

*SN*_*n*_*(TN*_*ij*_*) = argmax*_*sn*_ *DC(TN*_*ij*_*, sn)*,

where *sn* is any subnetwork enriched in Run *n*, and *DC(x, y)* indicates the dice coefficients between two subnetworks *x* and *y*. Consequently, for Run *j*, we define its *i*-th consensus module CM_ij_ as follows.

$$ C{M}_{ij}=T{N}_{ij}\cap \left(\bigcap \limits_{j=1,2,...\mathrm{10}}^{i=1,2,...\mathrm{5}}S{N}_n\left(T{N}_{ij}\right)\right),n\in \left\{1,\mathrm{2...10}\right\}\backslash \left\{j\right\} $$

Namely, *CM*_*ij*_ is the intersection of *TN*_*ij*_ and its most similar subnetworks identified in all the other runs. In our empirical study, we will report the consensus modules based on Run 1, i.e., *CM*_*i1*_ as the *i*-th consensus module.

### Functional analysis

Cytoscape 3.4 [40] was used to visualize the identified *CMs*. We used ToppGene online tool (https://toppgene.cchmc.org/) for functional enrichment analysis. The ToppGene suite is an advanced bioinformatics tool, it could detect and arrange candidate genes through a comprehensive assessment of a variety of factors, including gene ontology (GO) annotating, phenotype, signaling pathway and protein interactions from a specific list of genes [41]. In this case, the top 10 findings of our multivariate gene-based association analysis were analyzed for functional enrichment. For the identified CMs, we also performed functional enrichment analysis using the ToppGene Suite.

## Data Availability

The genotyping and subcortical imaging phenotypes data were downloaded from the Alzheimer’s Disease Neuroimaging Initiative (ADNI) database (http://adni.loni.usc.edu/). Application for access to the ADNI data can be submitted by anyone at http://adni.loni.usc.edu/data-samples/access-data/. The process includes completion of an online application form and acceptance of Data Use Agreement. We have received administrative approval for access to the ADNI database. The Human PPI data were downloaded from the public Human Protein Reference Database (http://www.hprd.org/).

## Abbreviations

AD: Alzheimer’s disease
QTs: Quantitative traits
GWAS: Genome-Wide Association Studies
QT: Quantitative trait
SNPs: Single Nucleotide Polymorphisms
AD: Alzheimer’s Disease
MGAS: Multivariate Gene-based Association test by extended Simes procedure
iPINBPA: Integrative Protein-Interaction-Network-Based Pathway Analysis
PPI: Protein-protein interaction
CM: Consensus Module
CMs: Consensus Modules
SNP: Single nucleotide polymorphism
SNPs: Single nucleotide polymorphisms
HPRD: Human Protein Reference Database
ADNI: Alzheimer’s Disease Neuroimaging Initiative
MRI: Magnetic resonance imaging
PET: Positron emission tomography
MCI: Mild cognitive impairmen
CN: Cognitively normal
SMC: Significant memory concern
EMCI: Early mild cognitive impairment
LMCI: Late mild cognitive impairment
ANOVA: Analysis of variance
DC: Dice’s coefficient
GNs: Groups of enriched subnetworks
TN: Top subnetwork.
